# Validation of the picoAMH assay on the Dynex DS2 platform

**DOI:** 10.1016/j.plabm.2019.e00140

**Published:** 2019-09-27

**Authors:** Emily R Garnett, Purviben Jariwala, Kesha Rector, William E Gibbons, Paul W Zarutskie, Sridevi Devaraj

**Affiliations:** a Department of Pathology & Immunology, Baylor College of Medicine, Houston, TX, 77030, USA; b Department of Obstetrics and Gynecology, Baylor College of Medicine, Houston, TX, 77030, USA

**Keywords:** Anti-Müllerian hormone, Infertility, Method validation, Ovarian reserve

## Abstract

**Objectives:**

Anti-Müllerian hormone (AMH) is increasingly used as a biomarker of ovarian reserve in clinical practice, and is used both for management of fertility treatments and prediction of menopause. We sought to validate the newly FDA-approved Ansh Laboratories MenoCheck picoAMH ELISA on the Dynex-DS2 platform for clinical use in our obstetrics and gynecology center.

**Design:**

Validation of the picoAMH ELISA on the Dynex-DS2 was performed according to CLSI guidelines. Intra- and inter-assay CV, assay linearity, and method comparison studies were carried out to verify assay precision and accuracy. The manufacturer’s reference range was verified using 26 volunteer samples, and interference for hemolysis, lipemia, icterus, and biotin was evaluated. picoAMH results were additionally correlated with antral follicle count by ultrasound.

**Results:**

Intra- and inter-assay CV of the picoAMH assay on the DS2 was ≤4% and the assay was linear between concentrations of 0.0067–16.24 ng/mL (0.048–116.0 pmol/L) AMH. Method comparison was performed with the manufacturer’s laboratory and indicated good correlation, with Deming regression yielding slope of 0.928 and intercept of −0.0421. The assay displayed no significant interference from hemolysis (1000 mg/dL), lipemia (2000 mg/dL), conjugated bilirubin (66 mg/dL), or biotin (10,000 ng/mL). Measurement of AMH on the DS2 was also correlated with antral follicle count, with *R* = 0.7128.

**Conclusions:**

Our results indicate that the picoAMH ELISA on the DS2 has good analytical performance suitable for clinical use.

## 1 Introduction

Anti-Müllerian hormone (AMH) is a homodimeric glycoprotein with roles in regulation of fetal sexual differentiation and folliculogenesis in the adult ovary. In adult women, AMH production is restricted to granulosa cells in growing ovarian follicles and is thought to be a regulator of dominant follicle selection [1]. Contemporary clinical interest in AMH is primarily in its utility as a marker for ovarian reserve, with an increasing number of laboratories offering measurement of AMH for prediction of response to controlled ovarian stimulation in assisted reproductive therapy [2].

The most common method of measurement used for AMH is enzyme-linked immunosorbent assay (ELISA), although earlier ELISA assays have suffered from problems with sample stability [3] and measurement variability [4]. Newer-generation assays have equivalent performance with fewer reproducibility issues [5,6]. A picoAMH assay with a lower detection threshold from Ansh Laboratories was first described in 2014, and other studies have highlighted its utility for detection of low levels of AMH [[7], [8], [9]]. As of 2018, a version of this assay is now FDA-approved for determination of menopausal status [10]. In this report, we describe the validation and characterization of the FDA-approved MenoCheck picoAMH ELISA from Ansh Laboratories using the Dynex-DS2 automated immunoassay system, and evaluate its utility for monitoring of ovarian reserve in a reproductive endocrinology and infertility/in vitro fertilization (REI/IVF) setting.

## 2 Materials and methods

The Ansh Laboratories picoAMH ELISA assay (Webster, TX) was evaluated for use on the Dynex-DS2 automated immunoassay system. The picoAMH assay yields measurements in pg/mL, and all measurements from the instrument were converted to standard units of ng/mL.

Intra- and inter-assay precision studies were performed in accordance with CLSI guidelines by measuring two levels of picoAMH quality control (QC) material (level 1 QC concentration of 0.079–0.131 ng/mL [0.564–0.935 pmol/L] and level 2 QC concentration of 0.258–0.388 ng/mL [1.84–2.77 pmol/L]). Intra-run precision was assessed by measurement of 10 replicates within one run, and inter-assay precision was assessed by measurement once a day for 20 days. CV was calculated for intra- and inter-assay studies by standard deviation/mean X 100, and results were considered acceptable when CV < 10%.

Linearity studies were carried out in accordance with CLSI guidelines by measuring 6 levels of picoAMH assay calibrators (with given AMH concentrations of 0.0067–1.091 ng/mL [0.0478–7.793 pmol/L]) in triplicate, and allowable systematic error set at 5.0%. Dilution verification studies were also carried out by testing six samples neat and with a 16X manual dilution. These samples were run in duplicate, and results considered acceptable if the difference from neat specimens was <10%. Recovery of AMH in diluted samples was calculated by:

$$Recovery\left(\%\right)=\frac{\left[diluted\;sample\right]}{\left[neat\;sample\right]}\times 100$$

Accuracy of the picoAMH ELISA on the Dynex-DS2 was determined by comparison with ELISA performed onsite at the manufacturer’s CLIA-certified laboratory (Ansh Laboratories). 28 serum samples were collected from patients for whom AMH testing was requested and were measured by both assays. Serum was separated within 20 minutes of specimen receipt, and all samples were stored frozen at −20 °C prior to analysis, then thawed just prior to analysis. AMH concentrations of the samples spanned the analytical measurement range of the assay.

A subset of the specimens used in accuracy studies were used to perform correlation of AMH measurements with antral follicle count (AFC). AFC was taken as the sum of antral follicles identified in right and left ovaries by ultrasound, and this was interpreted by faculty in reproductive endocrinology and infertility. Patients for whom AMH testing and AFC by ultrasound was performed on the same day, and with successful visualization of both ovaries (N = 12) were included in the correlation.

Reference ranges given by the manufacturer were verified using samples from healthy female volunteers aged 22–41 years (N = 26), with samples tested as previously described. The reference range was deemed acceptable if 95% of the healthy cohort fell within the given range.

Assay interferences were verified by spiking pooled serum samples (10 donors/pool) with the manufacturer-indicated concentrations of hemoglobin (1000 mg/dL), bilirubin (66 mg/dL), or intralipid (2000 mg/dL) using a commercial interference kit (Sun Diagnostics). Biotin interference was also verified by spiking with D-biotin (10,000 ng/mL) (Sigma-Aldrich). Samples were measured in triplicate, and results were considered acceptable if the difference from neat specimens was <15%.

Results from all studies were evaluated statistically in EP Evaluator. Method comparison was evaluated using Deming regression and Bland-Altman analysis. Paired *t*-test was performed on method comparison data sets, with p = 0.05 considered significant.

## 3 Results

The picoAMH ELISA demonstrated good intra-assay precision at both low and high levels, with CV 2.0% at a mean concentration of 0.101 ng/mL (0.728 pmol/L) AMH, and CV 2.0% at 0.315 ng/mL (2.25 pmol/L). Inter-assay precision was also good, with CV 4.4% at 0.313 ng/mL (2.24 pmol/L) and CV 3.6% at 0.893 ng/mL (6.38 pmol/L) AMH.

**Fig. 1:**
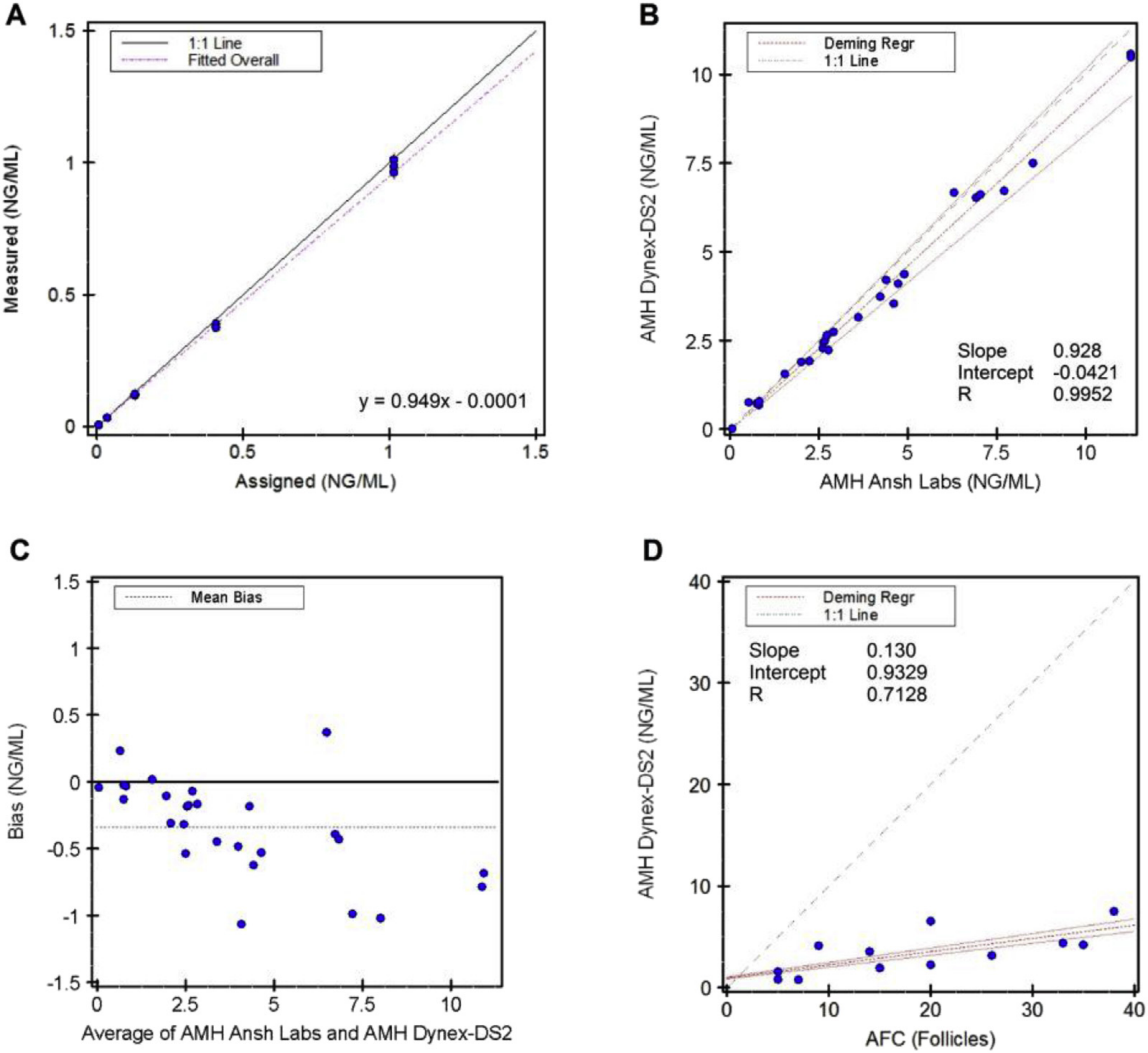
Validation studies for the picoAMH assay on Dynex-DS2, according to CLSI EP6 guidelines. **A:** Linearity of the picoAMH ELISA on Dynex-DS2. **B:** Deming regression analysis between AMH values measured by picoAMH on the Dynex-DS2 in our laboratory and the picoAMH at Ansh Laboratories. **C:** Bland-Altman analysis for method comparison between the Dynex-DS2 and Ansh Laboratories. Mean bias of the Dynex-DS2 relative to the manufacturer’s method was −0.348 ng/mL. **D:** Deming regression of picoAMH measurements by Dynex-DS2 and same-day antral follicle count (AFC) by ultrasound.

The picoAMH ELISA was found to be linear between 0.0067 and 1.015 ng/mL (0.0478–7.250 pmol/L), with total systematic error of 2.5% (Fig. 1A). Dilution studies indicated recovery of 98–108% with a 1:16 dilution, with all tested samples falling within 10% of the neat sample results. The clinical reportable range for the MenoCheck picoAMH was verified to be 0.0067–16.24 ng/mL (0.048–116.0 pmol/L).

Method comparison studies with the manufacturer’s laboratory indicated good agreement with the picoAMH ELISA on the Dynex-DS2. Deming regression yielded DS2 = 0.928 (Ansh Labs) – 0.0421 ng/mL. Pearson’s correlation coefficient (*R*) for the two assays was 0.9952, indicating very good concordance between assays (Fig. 1B). However, the Dynex-DS2 exhibited negative bias relative to ELISA performed at Ansh Labs, with a mean difference of −0.348 ng/mL (−2.48 pmol/L) (Fig. 1C). Paired *t*-test indicated this difference was significant (p < 0.01).

Reference range verification studies were carried out to verify reference intervals for AMH from Ansh Laboratories for their in-house picoAMH ELISA (0.401–16.015 ng/mL [2.864–114.4 pmol/L] for females ages 18–29 years, 0.176–11.705 ng/mL [1.257–83.61 pmol/L] for females ages 30–39 years) [11]. In our donor population, two specimens yielded values above the linear range of the assay and were excluded. The remainder of the measured values fell within the manufacturer’s reference interval, with values occupying a range of 1.202–11.816 ng/mL (8.586–84.40 pmol/L).

Interference studies were performed to examine the effect of common interferents on the picoAMH ELISA. No significant interference was observed with hemolysis, lipidemia, icterus, or biotin at high concentrations, with all compounds producing <10% difference from a neat sample (Table 1).

**Table 1:** Interference studies with the Dynex-DS2.

Interferent	Mean AMH, ng/mL (pmol/L)	% difference
Neat	2.13 (15.21)	–
Hemolysate 1000 mg/dL	1.96 (14.00)	−8.0%
Triglyceride-rich lipid 2000 mg/dL	2.08 (14.86)	−2.5%
Conjugated bilirubin 66 mg/dL	2.21 (15.79)	3.6%
D-biotin 10000 ng/mL	2.09 (14.93)	−2.0%

Comparison of same-day picoAMH measurement by Dynex-DS2 and AFC identified positive correlation between the two markers. Deming regression yielded AMH = 0.130 (AFC) – 0.9329. Pearson’s correlation coefficient (*R*) for the two assays was 0.7128 (Fig. 1D).

## 4 Discussion

Measurement of AMH is an emerging tool in the management of infertility and determination of ovarian reserve. We sought to validate the picoAMH ELISA for clinical use in our patient population at Texas Children’s Hospital, Pavilion for Women, REI/IVF unit. We find that the picoAMH assay performs well on the Dynex-DS2, with good precision and good agreement between our laboratory and the manufacturer.

The MenoCheck picoAMH ELISA has been FDA approved for determination of menopausal status. Previous generations of AMH assays have not been sensitive enough for this purpose, as the detection limit of these assays is not low enough to distinguish between patients >5 years and <5 years from their final menstrual period [12]. We have verified the linearity of this assay to 0.0067 ng/mL (0.0478 pmol/L), which is approximately 100-fold lower than the published limit of detection of the Ultrasensitive AMH Assay, also from Ansh Laboratories [7]. Limit of detection studies have not been performed for the MenoCheck assay, but other studies of the picoAMH assay have indicated LoD of 0.0018 ng/mL (0.0129 pmol/L) and LoQ of 0.0030 ng/mL (0.0214 pmol/L) [8].

Our precision studies indicate that the picoAMH assay on the Dynex-DS2 has excellent reproducibility, with CV ≤ 4.4% both within and between runs. The performance of the assay site-to-site also is good. We observed excellent correlation between the Dynex-DS2 at our laboratory and the manufacturer’s assay (Fig. 1B). We did observe small but significant negative bias (−0.348 ng/mL [-2.48 pmol/L]) of the Dynex-DS2 relative to the manufacturer’s laboratory (Fig. 1C). The differences in measurements observed in our laboratory as compared to the manufacturer may be attributable to different sample storage times, and freeze-thaw cycle, as AMH levels have been reported to increase with storage at −20 °C [13].

The assay is also robust to common sample interferences (Table 1). These studies have not been reported in other method validation studies, and this information is an important addition to the literature on the picoAMH assay. The assay manufacturer indicates that mature AMH (prior to undergoing glycosylation and dimerization) exhibits only 0.217% crossreactivity with the picoAMH assay, with no other proteins exhibiting significant crossreactivity with the assay [11]. However, AMH is homologous to transforming growth factor beta (TGFβ), and the picoAMH assay literature does not specify crossreactivity with this protein.

Little literature is available on cross-reactivity with other AMH assays. Per the manufacturer, the Beckman Access AMH enzyme immunoassay has no crossreactivity with TGFβ [15]. Other crossreactivity information is not reported by the manufacturer for the Beckman Gen II AMH ELISA or the Roche Elecsys AMH Immunoassay. However, the Beckman Gen II ELISA has been reported to suffer interference from complement, while automated AMH assays from Beckman and Roche, which are standardized to the Gen II ELISA, do not [14]. Future studies could explore whether complement interference is a concern in the picoAMH assay, given that both the Gen II and picoAMH assays rely on ELISA methodology.

We were able to verify the manufacturer’s reference interval for ages 18–29, with 100% of our measured values falling within the Ansh Laboratories interval for this age range [11]. However, we collected specimens from an insufficient number of donors to independently verify the reference interval for individuals aged 30–39 years (N = 12). There are no published studies identifying reference ranges for the picoAMH ELISA to which we could compare our results. Overall, there is a paucity of other reference range and clinical correlation data available for AMH, which may be partially driven by the lack of standardization between AMH assays [15].

Measurement of AFC by ultrasound is also widely used as a marker of ovarian reserve and has been reported to correlate well with AMH measurements [16]. We found that measurement of AMH on the Dynex-DS2 correlates positively with measurement of AFC (Fig. 1D). Although menstrual cycle day varied by individual, AMH and AFC measurements were performed on the same day, which permits comparison. A major limitation of AFC is variability and subjectivity intrinsic to the measurement process [17]. Measurement of AMH along with AFC could offer an opportunity to improve objectivity and accuracy of ovarian reserve estimation.

Our study is limited by the fact that we did not investigate diagnostic cutoff values for assay of AMH by picoAMH. Studies to establish cutoff values for AMH will be ongoing at our institution. In addition, the utility of an algorithm incorporating both AMH and AFC will be explored once sufficient data is obtained.

The ability to measure AMH at low concentrations is a promising tool for evaluation of ovarian reserve, and will likely be useful in monitoring of fertility treatments or chemotherapy that affects patients’ fertility status. However, a major limitation of AMH assays remains the absence of a standard reference material and subsequent lack of standardization of assays [15]. The Beckman Gen II AMH ELISA has been widely used, but has been redeveloped since its introduction due to performance issues with the original version [3,4]. Automated AMH assays have also been introduced by Beckman (the Access AMH assay) and Roche (Elecsys AMH Immunoassay). While other reports demonstrate generally good correlation among these and the Ansh Laboratories methods, the calibrators for these assays differ, which may partially explain the published numerical differences between methods [8,15]. Standardization of AMH measurements will be required to allow for generation of widely applicable reference ranges and clinical decision limits for evaluation of patients.

### 4.1 Conclusions

This study describes the assay characteristics of the picoAMH assay on the Dynex DS2 platform. We have shown that it can be used for patient testing in the REI/IVF population in our hospital.

## Declaration of competing interest

Authors report no conflicts of interest. E.G was supported by the Ching Nan Ou Fellowship in Clinical Chemistry.
